# Candidate Alzheimer’s Disease Biomarker miR-483-5p Lowers TAU Phosphorylation by Direct ERK1/2 Repression

**DOI:** 10.3390/ijms22073653

**Published:** 2021-04-01

**Authors:** Siranjeevi Nagaraj, Andrew Want, Katarzyna Laskowska-Kaszub, Aleksandra Fesiuk, Sara Vaz, Elsa Logarinho, Urszula Wojda

**Affiliations:** 1 Laboratory of Preclinical Testing of Higher Standard, Nencki Institute of Experimental Biology of Polish Academy of Sciences, Pasteur 3, 02-093 Warsaw, Poland; s.nagaraj@nencki.edu.pl (S.N.); a.want@nencki.edu.pl (A.W.); k.laskowska-kaszub@nencki.edu.pl (K.L.-K.); a.fesiuk@nencki.edu.pl (A.F.); 2 i3S, Institute for Research and Innovation in Health, University of Porto, 4200-135 Porto, Portugal; sara.vaz@ibmc.up.pt (S.V.); elsa.logarinho@ibmc.up.pt (E.L.); 3 Aging and Aneuploidy Laboratory, IBMC, Institute of Molecular and Cellular Biology, University of Porto, 4200-135 Porto, Portugal

**Keywords:** Alzheimer’s disease, microRNA, extracellular signal-regulated kinases (ERK), microtubule associated protein TAU (MAPT)

## Abstract

MicroRNAs have been demonstrated as key regulators of gene expression in the etiology of a range of diseases including Alzheimer’s disease (AD). Recently, we identified miR-483-5p as the most upregulated miRNA amongst a panel of miRNAs in blood plasma specific to prodromal, early-stage Alzheimer’s disease patients. Here, we investigated the functional role of miR-483-5p in AD pathology. Using TargetScan and miRTarBase, we identified the microtubule-associated protein MAPT, often referred to as TAU, and the extracellular signal-regulated kinases 1 and 2 (ERK1 and ERK2), known to phosphorylate TAU, as predicted direct targets of miR-483-5p. Employing several functional assays, we found that miR-483-5p regulates ERK1 and ERK2 at both mRNA and protein levels, resulting in lower levels of phosphorylated forms of both kinases. Moreover, miR-483-5p-mediated repression of ERK1/2 resulted in reduced phosphorylation of TAU protein at epitopes associated with TAU neurofibrillary pathology in AD. These results indicate that upregulation of miR-483-5p can decrease phosphorylation of TAU via ERK pathway, representing a compensatory neuroprotective mechanism in AD pathology. This miR-483-5p/ERK1/TAU axis thus represents a novel target for intervention in AD.

## 1. Introduction

Alzheimer’s disease (AD) is an ageing-related, progressive and irreversible neurodegenerative disorder accounting for the majority of dementia cases in the elderly. It is characterized by memory loss, cognitive decline and difficulties in communication along with marked behavioral changes. In 2015 around 47 million people were reported to suffer from AD-related dementia, and this number is forecasted to reach 131.5 million by 2050 [1]. Due to this high prevalence and the resulting generation of a huge socioeconomic burden, AD is one of the major challenges in the societies of the 21st century [2].

The main neuropathological AD features are: extracellular senile plaques made up of aggregates of amyloid beta (Aβ) peptides, and intracellular deposits of hyperphosphorylated TAU protein, known as neurofibrillary tangles (NFTs) [3,4]. The alterations in the AD brain develop gradually for years; the period of dormancy between disease onset at the molecular level and the manifestation of the clinical symptoms, named preclinical AD, usually lasts more than a decade. The preclinical stage is usually followed by Mild Cognitive Impairment (MCI) due to AD (prodromal AD) and later progresses from mild to severe AD [5]. Furthermore, the pathogenesis and development of AD is dependent on many genetic and environmental factors which are not fully elucidated [6]. This results in a lack of disease-modifying therapies for AD despite multiple clinical trials with a variety of potential drugs (www.clinicaltrials.gov, last accessed: 10 March 2021). Another unmet need in AD is the availability of broadly accessible, non-invasive diagnostic methods for detection of disease, prior to the onset of symptoms. It is clear that the critical step towards the design of effective diagnostic tests and therapeutic strategies is to improve understanding of the early molecular mechanisms of AD. 

Recently, we and others have shown that molecular alterations in AD are not restricted to the brain but can be detected in peripheral tissues such as blood, opening the possibility for the identification of easy-to access, early AD biomarkers. Of particular interest are microRNAs (miRNAs); short non-coding RNA molecules (19–24 nucleotides) and crucial epigenetic regulators of more than 60% of human genes at the post-transcriptional level. Usually, miRNA binds mRNA in the 3′-untranslated region (3′-UTR) inducing mRNA degradation or translational inhibition [7]. microRNAs play crucial roles in epigenetic regulation of the genes involved in AD pathology affecting processes such as amyloid-β accumulation and TAU hyperphosphorylation in the AD brain [8,9,10,11]. In addition to their effects within the brain, the increase in blood-brain barrier permeability associated with AD enables greater exchange of brain-resident miRNAs with blood and vice versa [12]. This increases the likelihood that molecules proximal to the AD pathomechanism will appear in the blood, as well as that normally excluded miRNAs will reach the brain and modulate AD. Moreover, miRNAs are often secreted to blood plasma in exosomes, where they are more stable, regulating distant cells in a hormone-like fashion [13]. 

Evidence gathered from several studies has indicated that multiple miRNAs are differentially expressed in blood from AD patients compared to non-demented subjects, as reviewed [14,15,16]. Recently, we reported a panel of 15 miRNAs whose levels were altered in the blood plasma of AD patients and also of prodromal AD patients, i.e., an early stage of AD documented based on the cerebrospinal fluid (CSF) levels of Aβ_42_, TAU and phospho-TAU [17]. These miRNAs are very promising as early, low-interventional AD biomarkers. The most significantly increased miRNA in AD blood was miR-483-5p (14-fold increase over control, Receiver operating characteristic (ROC) parameters: Area under the curve (AUC) > 0.92, specificity = 1, sensitivity > 0.8) [17]. As currently little is known on the miR-483-5p regulatory function in AD pathology, in this study we addressed this question. 

## 2. Results

### 2.1. miR-483-5p Predicted to Target TAU, ERK1 and ERK2 Involved in Neurofibrillary Tangles (NFTs) Pathology in AD

**Figure 1:**
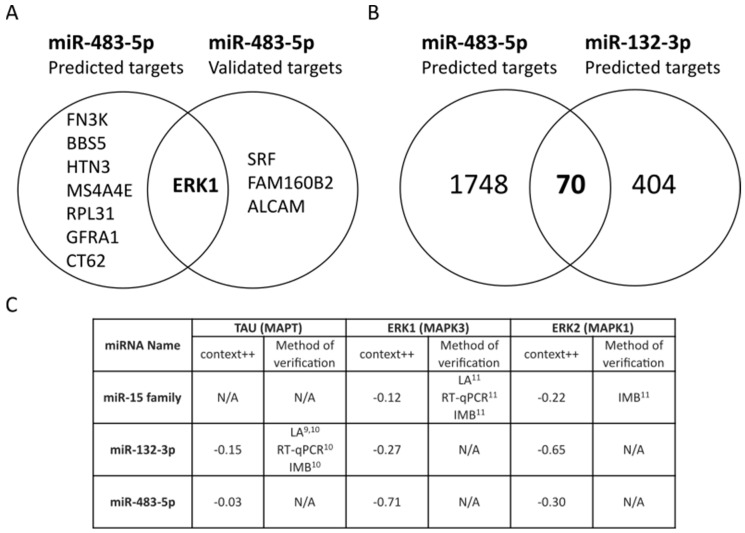
miR-483-5p predicted binding to mRNAs from ERK1 (*MAPK3*) and ERK2 (*MAPK1*). (**A**) Top 8 out of 1818 predicted targets of miR-483-5p from TargetScan 7.2 (based on context++ score) and the overlap with four experimentally-validated targets of miR-483-5p in miRTarBase 7.0. (**B**) Common targets predicted from TargetScan 7.2 for miR-483-5p and the neuroprotective miR-132-3p. (**C**) Table of predicted miRNA affinities for TAU (*MAPT*), ERK1 (*MAPK3*) and ERK2 (*MAPK1*) mRNAs in AD literature. LA = Luciferase assay, RT-qPCR = Reverse transcriptase quantitative PCR, IMB = Immunoblotting, N/A = Not available. The superscripted numbers indicate references where these interactions were verified.

We found 1818 predicted targets using TargetScan (v7.2) [18], and four experimentally validated targets using miRTarBase (v7.0) [19], for miR-483-5p. Based on TargetScan context++ scores, ERK1 mRNA has the seventh strongest predicted interaction of the 1818 predicted targets for miR-483-5p and is the only target in the top eight with experimental validation (Figure 1A). miR-132-3p is one of the most studied miRNAs for its role in neuroprotection in Alzheimer’s disease models [10,20,21]. There is a consensus that cellular upregulation of miR-132-3p ameliorates the neurodegeneration process by repressing several targets involved in complex neurodegenerative pathways [22,23,24,25]. When comparing miR-483-5p with the neuroprotective miR-132-3p, we found 70 common predicted targets (Figure 1B). The context++ scores of these common targets are plotted in Supplementary Figure S1 for reference. Three key potential targets are illustrated in Figure 1C; TAU (*MAPT*) for its integral association with Alzheimer’s disease neurodegeneration, ERK1 (*MAPK3*) already demonstrated to be regulated by miR-483-5p and ERK2 (*MAPK1*) for its similarity to ERK1 and the promising context++ score. Alongside these, we have also highlighted the miR-15 family (miR-15a-5p, miR-15b-5p, miR-16-5p, miR-195-5p and miR-497-5p) which was previously shown to regulate ERK1 in AD [11]. The most favorable predicted interaction is between miR-483-5p and ERK1 (context++ score: −0.71), with experimental validation in a small number of in vitro systems [26,27,28], although still functionally elusive. The context++ scores of miR-483-5p are similar to those of miR-132-3p, hinting that the presence of miR-483-5p in the blood of AD patients might be also involved in neuroprotective activity. Furthermore, there is potential for a direct interaction between miR-483-5p and *MAPT* transcript, despite the low context++ score (−0.03). 

### 2.2. miR-483-5p Does Not Bind to TAU mRNA

**Figure 2:**
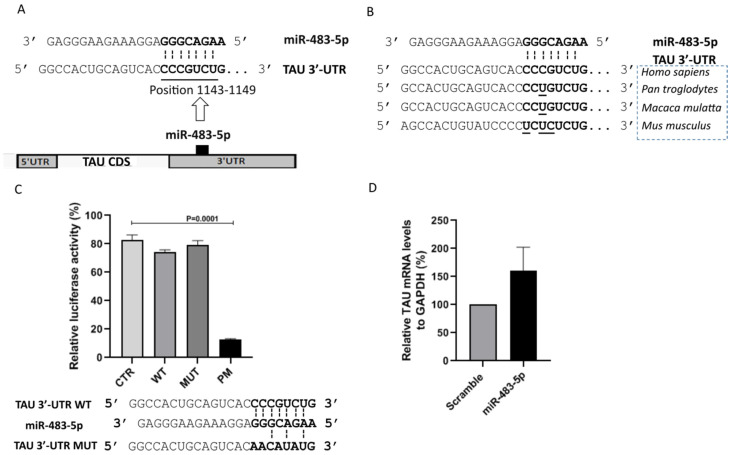
miR-483-5p does not directly bind to TAU mRNA or impact TAU levels. (**A**) Scheme represents the predicted binding site for miR-483-5p in the 3′-UTR of the TAU mRNA. (**B**) Prediction indicates human-specific miR-483-5p binding site in 3′-untranslated region (3′-UTR) of the TAU mRNA among different species. (**C**) Quantitative reporter assay for luciferase activity in HEK293 cells. Reporter constructs carrying a single binding site (CTR, WT, MUT, PM) for miR-483-5p were tested. The miRNA activity on four pmirGLO constructs was measured simultaneously: an empty pmirGLO vector (CTR), a wild-type potential binding site for miR-483-5p (WT), a mutated binding site (MUT), and a site with full complementarity (PM). Interaction scheme of WT and MUT binding site of 3′-UTR of the TAU mRNA with miR-483-5p are shown below. PM (not shown here) is designed to perfectly match the miR-483-5p. Firefly luciferase activity was normalized against renilla luciferase activity. Data are shown as mean ± standard error from four independent experiments. One way ANOVA with post-hoc Tukey HSD was used for statistical analysis. (**D**) RT-qPCR analysis of TAU mRNA levels upon miR-483-5p mimic transfection in SK-N-MC cells. GAPDH was used as a reference. Data are shown as mean ± standard error from three biological replicates; Unpaired Student’s *t*-test was used for statistical analysis. In all cases, annotations were made on plots only where significant differences (*p* < 0.05) were found.

Towards assessing the putative role of miR-483-5p in AD, we first probed for a direct interaction with TAU, one of the two major hallmarks of AD. The interaction between miR-483-5p and TAU 3′-UTR (at the position 1143-1149) is a poorly conserved 7mer-m8 site type (exact match to positions 2–8 of the mature miRNA) with the context++ score −0.03 (Figure 2A). Despite this low context++ score, the interaction is human-specific when analyzed across different species (Figure 2B). This observation prompted us to experimentally validate the predicted interaction using reporter constructs in luciferase assays. Figure 2C shows that only a perfect match between the TAU 3′-UTR and miR-483-5p results in any significant reduction in TAU mRNA in HEK293 cells. This finding was recapitulated in SK-N-MC cells with 100 nM miR-483-5p mimic transfection, where we observed no significant difference in the TAU mRNA levels compared to the scramble control (Figure 2D). Taken together, these experiments show that the interaction of miR-483-5p with TAU causes negligible direct impact on TAU mRNA (*MAPT*) expression.

### 2.3. miR-483-5p Binds to ERK1 mRNA and Represses Its Expression in HEK293 Cells

**Figure 3:**
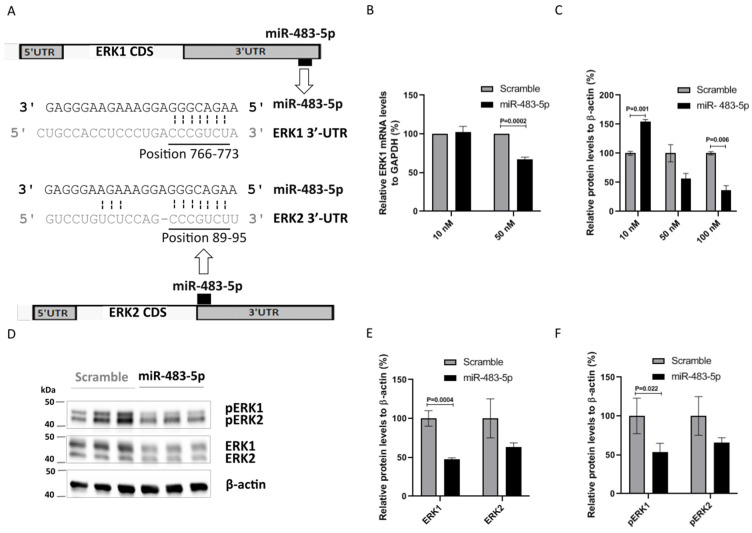
miR-483-5p downregulates ERK1 at both the mRNA and protein level in HEK293 cells. (**A**) Scheme representing the predicted binding site for miR-483-5p in ERK1 and ERK2 3′-UTRs. (**B**) RT-qPCR analysis of ERK1 mRNA levels upon transfection with 10 nM and 50 nM of miR-483-5p mimic. GAPDH was used for normalization. (**C**) Immunoblot quantitative analysis of ERK1 protein levels upon transfection with miR-483-5p mimic at different concentrations. β-actin levels were used for normalization. (**D**) Representative immunoblot for ERK1/2 and pERK1/2 protein levels upon transfection with scramble and miR-483-5p mimic at 100 nM. (**E**,**F**) Immunoblot densitometry analysis of ERK1/2 and pERK1/2 protein levels upon transfection with scramble and miR-483-5p mimic at 100 nM. Data are shown as mean ± standard error from three independent experiments; Unpaired Student’s *t*-test was used for statistical analysis.

Using TargetScan, we found that the interaction between miR-483-5p and ERK1 3′-UTR (at position 766–773) is a poorly conserved 8mer type site with a context++ score of −0.71 (Figure 3A). According to the miRmine database [29], HEK293 cells do not express miR-483-5p, thus positioning them as a naive cell model for miR-483-5p mimic transfection functional studies. We used scramble as a baseline control for evaluating the effect of miR-483-5p mimic. At 50 nM, miR-483-5p mimic significantly reduced ERK1 mRNA levels in comparison to scramble (Figure 3B). A reduction in ERK1 protein levels was also observed, although only statistically significant in comparison to scramble at 100 nM of miR-483-5p mimic (Figure 3C,D). We also note here that lower concentrations of miR-483-5p show a pronounced increase in ERK1 protein levels. We have observed this behavior in other protein systems with sub-inhibitory concentrations of miRNA; it is yet to be fully understood, however we believe that this may be the result of miR-483-5p interacting with the wider network, cumulatively enhancing ERK1 production without any direct effect. Moreover, TargetScan analysis revealed the interaction between miR-483-5p and the ERK1 homolog ERK2 at the 3′-UTR positions 89–95 to be a 7mer-m8 site type with a context++ score −0.30 (Figure 3A). Using antibodies against total and phosphorylated ERK1/2 proteins in HEK293 cell lysates (Figure 3D–F) we found that miR-483-5p mimic at 100 nM reduced both total and phosphorylated ERK1 and ERK2 protein levels in comparison to scramble control (Figure 3C–F), it was although statistically significant only for ERK1/pERK1. Overall, the results suggest that miR-483-5p inhibits the expression of ERK1, and less extensively ERK2, leading to decreased levels of pERK1/2.

### 2.4. CRISPR/Cas9-Mediated Genomic Deletion of miR-483-5p from Neonatal Fibroblasts Increases ERK1 mRNA Levels

**Figure 4:**
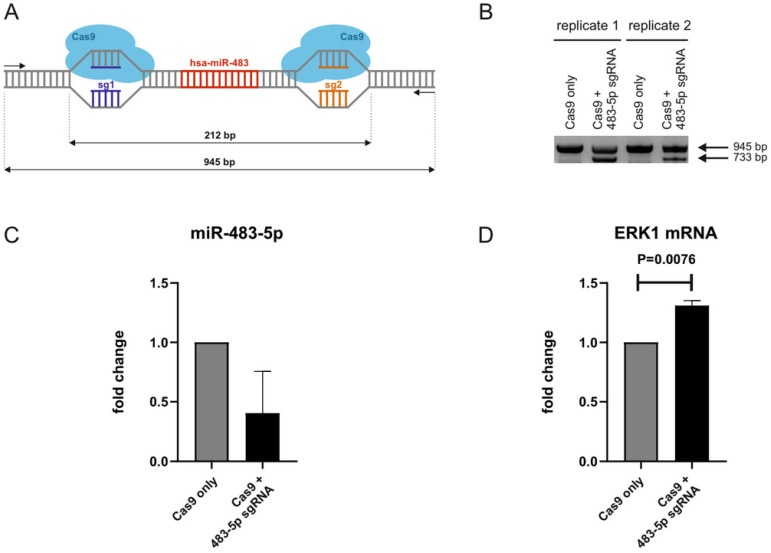
CRISPR/Cas9-mediated knockout of miR-483-5p. (**A**) Scheme of the CRISPR/Cas9-mediated genomic deletion of miR-483-5p using upstream and downstream sgRNAs (sg1 and sg2). Fragment sizes of the wild-type allele (945 bp) and deleted region (212 bp). (**B**) Agarose gel electrophoresis of PCR amplified fragments (using primers shown in **A**) from polyclonal cell populations retrieved upon transfection with empty plasmid without sgRNA (Cas9 only) and Cas9 +/sgRNA expressing plasmid (Cas9 + miR-483-5p sgRNA). 733 bp band corresponds to deleted allele. (**C**) Fold change decrease in miR-483-5p levels in CRISPR/Cas9-deleted cells (Cas9 + miR-483-5p sgRNAmiR-483 KO). miR-191-5p was used for normalization. (**D**) Fold change increase in ERK1 mRNA (*MAPK3*) levels in CRISPR/Cas9-deleted cells. GAPDH was used for normalization. Data represent the mean ± standard deviation from two independent experiments. Statistical analysis was performed using an unpaired Student’s *t*-test on log 2 (fold change).

To further investigate miR-483-5p interactions with ERK1 mRNA (MAPK3), we resorted to CRISPR/Cas9-mediated genomic deletion of miR-483-5p in neonatal fibroblasts. sgRNAs were designed to induce upstream and downstream cleavages in miR-483 coding sequence so that both 5p and 3p isomirs are deleted (Figure 4A). An empty plasmid (without sgRNA) was used as a control to exclude any toxicity due to Cas9. Efficient deletion was confirmed by gel electrophoresis to detect the presence of a 733 bp band in comparison to a 945 bp band corresponding to the wild-type allele (Figure 4B). Note that polyclonal populations consisting of cells knocked-out for miR-483-5p and cells still expressing miR-483-5p have been used for further analyses. Nevertheless, RT-qPCR analysis of miR-483-5p levels in the polyclonal populations revealed a deletion efficiency of ~70% (Figure 4C). Finally, we measured ERK1/*MAPK3* mRNA levels by RT-qPCR and found, as expected, increased expression (Figure 4D). These results further support that miR-483-5p binds to, and negatively regulates, ERK1.

### 2.5. miR-483-5p-Mediated Reduction in pERK1/2 Decreases pTAU in HEK293 and SK-N-MC Cells

**Figure 5:**
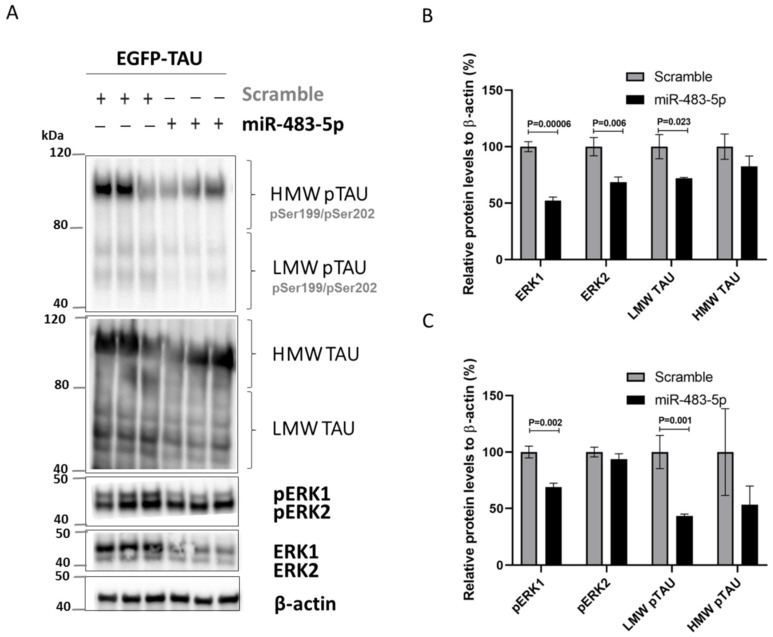
miR-483-5p mediated downregulation of total and phosphorylated ERK1/2 correlates with reduced levels of phosphorylated TAU in HEK293 cells expressing EGFP-TAU and upon transfection with scramble and miR-483-5p mimics at 100 nM. (**A**) Representative immunoblots of total and phosphorylated levels of TAU, ERK1 and ERK2 under the different experimental conditions. β-actin was used as loading control. (**B**) Quantitative analysis of the immunoblot levels for TAU, ERK1 and ERK2. (**C**) Quantitative analysis of the immunoblot levels for pTAU, pERK1 and pERK2. Data are shown as mean ± standard error of three independent experiments. One way ANOVA with post-hoc Tukey HSD was used and statistically significant differences are shown with *p* values.

Chronic activation of ERK1/2 can lead to neurodegeneration [30]. ERK1/2 appears to phosphorylate TAU [11,22], and pERK1/2 also regulates TAU expression via the Fyn/ERK/S6 pathway translational mechanism [31]. We therefore asked whether miR-483-5p-mediated reduction in phosphorylated ERK1/2 inhibits TAU phosphorylation, expression, or both in HEK293 cells overexpressing TAU. Indeed, we found significantly reduced levels of low molecular weight TAU (LMW TAU; 40–80 kDa) and its phosphorylated form (LMW pTAU) in cells transfected with 100 nM miR-483-5p mimic vs. scramble control (Figure 5A–C). High molecular weight TAU (HMW TAU; 80–120 kDa) and its phosphorylated form (HMW pTAU) levels were also reduced, although not significantly.

**Figure 6:**
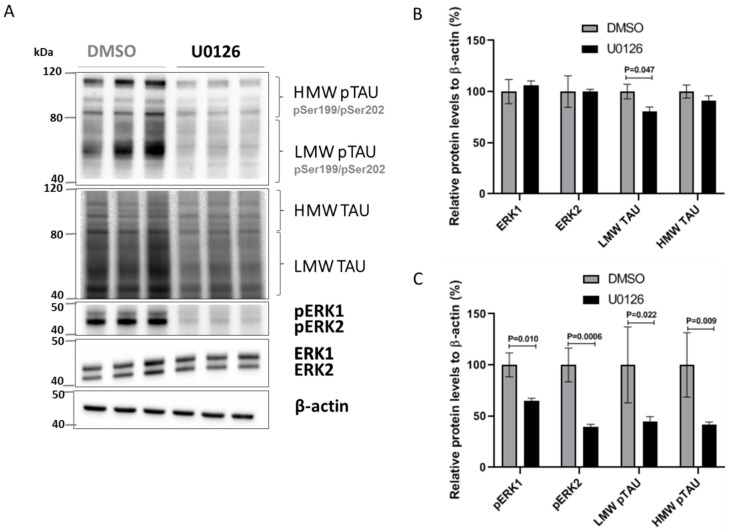
U0126 inhibition of ERK1/2 phosphorylation decreases total and phosphorylated TAU levels in SK-N-MC cells. (**A**) Representative immunoblots of total and phosphorylated levels of TAU, ERK1 and ERK2 in SK-N-MC cells treated with vehicle (DMSO) or with 10 µM of U0126. β-actin was used as loading control. (**B**) Quantitative analysis of the immunoblot levels for TAU, ERK1 and ERK2. (**C**) Quantitative analysis of the immunoblot levels for pTAU, pERK1 and pERK2. Data are shown as mean ± standard error of the mean from three independent experiments; Unpaired Student’s t-test was used to compare groups and statistically significant differences shown with *p* values.

To further validate this ERK/TAU axis, we used an alternative approach to reduce phosphorylated ERK1/2: U0126, an inhibitor for the MEK1/2 kinases that phosphorylate ERK1/2 (Figure 6A). SK-N-MC cells treated with U0126 exhibited a marked decrease in pERK1/2, LMW TAU and LMW pTAU (Figure 6A–C).

**Figure 7:**
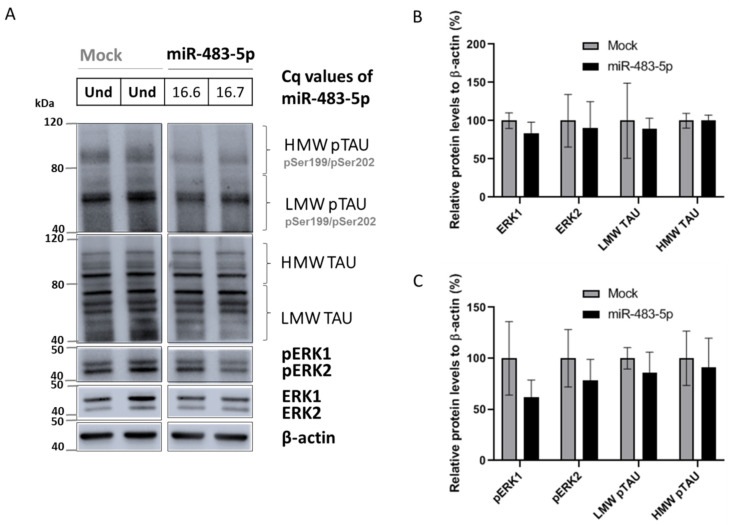
miR-483-5p-mediated downregulation of phosphorylated ERK1/2 is associated with a trend in decreased levels of phosphorylated TAU in SK-N-MC cells. (**A**) Representative immunoblots of total and phosphorylated levels of TAU, ERK1 and ERK2 in untransfected (mock) or transfected SK-N-MC cells with 100 nM miR-483-5p mimic. C_q_ values for miR-483-5p transfected cells are shown and in mock miR-483-5p was undetermined (Und). β-actin was used as loading control. (**B**) Quantitative analysis of the immunoblot levels for TAU, ERK1 and ERK2. (**C**) Quantitative analysis of the immunoblot levels for pTAU, pERK1 and pERK2. Data are shown as mean ± standard error of the mean from four biological replicates; Unpaired Student’s *t*-test was used to compare groups and we observed no statistically significant differences.

In addition, we transfected SK-N-MC neuronal cells with miR-483-5p (Figure 7A). miR-483-5p mimic at 100 nM, reduced ERK1/2, pERK1/2, LMW TAU, LMW pTAU, HMW TAU and HMW pTAU, although the reductions were not statistically significant in comparison to mock control (Figure 7A–C). Still, the trend in results was similar to that in HEK293 cells (Figure 5A–C). Overall, our results show that miR-483-5p inhibits TAU protein phosphorylation and its expression via repression of pERK1/2 activity.

## 3. Discussion

miRNAs are emerging as important post-transcriptional regulators of mRNA in both physiological and pathological processes, but little is known about their role in modifying disease pathways in AD. We previously identified a panel of 15 miRNAs in blood plasma with diagnostic potential for AD and for prodromal AD in MCI patients [17]. This study is a direct follow up, exploring the functional role of miR-483-5p, the most upregulated miRNA in the 15 miRNAs panel. In the current study, using an in silico approach, we identified that miR-483-5p is predicted to bind the 3′-UTR of mRNAs encoding ERK1, ERK2 and MAPT, all known to be involved in AD pathology. In HEK293 cells, using a range of experimental methods including luciferase assays, RT-qPCR and immunoblotting we found substantial evidence that ERK1 is strongly downregulated by miR-483-5p by direct interaction between the miRNA and the ERK1 mRNA transcript. CRISPR/Cas9-mediated genomic deletion of miR-483 in human primary neonatal fibroblasts provided supporting evidence for increased ERK1 (*MAPK3*) mRNA in comparison to wild-type control. Moreover, our study revealed weaker regulation of ERK2 and no direct binding or regulation of MAPT by miR-483-5p. In HEK293 cells transfected with an EGFP-TAU plasmid, we observed miR-483-5p-mediated downregulation of ERK1, and to a lesser extent ERK2. This was associated with downregulation of phosphorylated LMW pTAU, but less-so with aggregation-prone HMW pTAU, at the epitopes associated with neurofibrillary tangle pathology in AD. We observed this trend in the downregulation of ERK1/2 and pTAU in neuronal SK-N-MC cells transfected with miR-483-5p mimic. The observed changes in neuroblastoma cells did not reach statistical significance which could be attributed to two factors: (1) cell type-specific effects—different levels of available mRNAs capable of regulation by miR-483-5p between HEK293 and SK-N-MC, with more targets present in neuroblastoma cells; (2) technical issues—difference in the transfection efficiency; lower efficiency is usually achieved in neuroblastoma cells. Nevertheless, alternative experiments employing the ERK1/2 kinase inhibitor U0126 validated ERK1/2 as a kinase phosphorylating TAU in SK-N-MC. Taken together, this study identified the existence of a miR-483-5p/ERK1/TAU axis which appears relevant for the elucidation of the complex molecular mechanisms in AD pathology. 

The capacity of miR-483-5p to counteract TAU pathology by blocking ERK1/2-mediated TAU phosphorylation is contextualized by the enrichment of miR-483-5p in the brain [32] and in still higher quantities in AD patients [17]. Combined with our previous data [17], these findings indicate that during the course of AD, miR-483-5p expression becomes elevated as a protective mechanism against the pathology. Similarly, neuroprotective miRNAs in AD pathology have been identified in the adult forebrain and hippocampus in *DICER* knockout mouse brains [11]. The loss of this type III RNAse, which processes precursor pre-miRNAs into mature miRNAs, resulted in pathological hyperphosphorylation of TAU. Hébert et al. identified ERK1/2 as one of the kinases mediating TAU hyperphosphorylation and showed its increased levels in the DICER KO brains, along with a decrease in miR-15 family members, which were also shown to regulate ERK1/2 expression and TAU phosphorylation. Thus, our results add to the evidence that there is a network of miRNAs including miR-483-5p and miR-15 family members which perform a protective function against TAU phosphorylation when upregulated, with their depletion allowing concomitant increase in pathological TAU phosphorylation and neurofibrillary tangle formation in the AD brain. 

The neuroprotective function of miR-483-5p is consistent with its co-expression with the insulin growth factor 2 gene (*IGF2*), being located within the second intron of *IGF2* at chromosome 11 [32,33,34]. While IGF2 plays a major role as a potent metabolic regulator in fetal development [35], increased levels of IGF2 were found in cerebrospinal fluid of AD patients [36,37]. IGF2 upregulation was shown to promote neurogenesis and synapse formation and represents a neuroprotective response against oxidative stress in human neurons [38,39,40,41,42]. Studies show not only co-expression of miR-483-5p with IGF2 [34] but also a specific role of miR-483-5p in induction of *IGF2* transcription [43]. These data point to a dual neuroprotective role of miR-483-5p: as an inhibitor of ERK1-mediated phosphorylation of TAU and as enhancer of IGF2 expression in a positive feedback loop. 

Furthermore, upregulation of miR-483-5p together with IGF2 is connected with the responsiveness of *IGF2* to NFκB transcription factor, which in the human nervous system is a key player in neuroprotection, mediating responses to various stress factors and being involved in inflammatory reactions [42,44]. The correlation between miR-483-5p expression and IGF2/NFkB points to miR-483-5p function at the interface of metabolism and inflammation. Mounting evidence demonstrates that metabolic impairment, systemic inflammation and neuroinflammation are interconnected and significantly contribute to neurodegeneration in AD [45,46]. These processes progress with ageing and are potentiated by other known AD risk factors such as bad diet, obesity and type 2 diabetes [47,48]. Moreover, both NFkB and IGF2 are expressed in many peripheral cell types including immune cells, which could additionally account for the increased level of miR-483-5p in blood plasma in AD. Given the blood-brain barrier (BBB) impairment in AD, circulating miRNAs such as miR-483-5p can cross the BBB in both directions, to and from the brain, contributing to the cross-talk between the periphery and brain in the pathology [14]. 

Progressive, low-grade, systemic inflammation and inflammatory neurodegeneration is known to be linked with a complicated network of miRNAs and their target mRNAs, out of which some miRNAs play a promoting role [49]. In contrast, the miR-483-5p activity reported here seems to represent neuroprotective/compensatory mechanisms in AD. Thus, our findings support the hypothesis that in the miRNA regulatory network some miRNAs promote neurodegeneration while others counteract the pathology.

Several studies have shown that miR-132-3p is neuroprotective generally [21,24,50,51] as well as specifically in AD [22,52]. Interrogation of the TargetScan database showed that mRNAs encoding ERK1, ERK2 and TAU are also theoretical targets of miR-132-3p, with similar context++ scores to miR-483-5p. There is also empirical demonstration that miR-132-3p interacts with ERK2 leading to modifications in synaptic plasticity [53] and in *ITPKB* mRNA which is upstream of ERK1/2 [22]. These data imply that there is significant overlap between the potential targets of miR-483-5p and miR-132-3p, suggestive of either redundancy or synergy.

Understanding the mechanisms behind altered expression of miRNAs in AD patients supports prospects for their use as disease biomarkers and as therapeutic targets. Disease-modifying therapies targeting miRNAs can be developed using two main approaches: (i) increasing protective transcript levels by silencing miRNAs responsible for their repression [54] or (ii) decreasing the harmful transcripts through mimicking of existing miRNAs [55] or designing inhibitory miRNAs for a specific mRNA. Recent progress has been made in the delivery technology of oligonucleotides to specific tissues, cells and subcellular compartments, including traversing the BBB for brain delivery. However, a major difficulty to overcome is the off-target effect due to promiscuous binding of a miRNA to many mRNAs. To circumvent this, optimized bioinformatics tools for the prediction of miRNA target transcripts and their experimental verification is essential. 

It is well-understood that miRNAs have the capacity to perform widespread post-transcriptional control of gene expression through prevention of translation, or enhancing degradation, of mRNA [56]. Despite this, only a small proportion of the possible targets of miRNA repression have been experimentally validated [57]. There are a number of publicly available tools that enable researchers to estimate the probability of a miRNA of interest binding to a specified mRNA target (or targets), although these vary in their concordance with experiments. We used one of the most widely reported tools, TargetScan (current version 7.2) (Whitehead Institute for Medical Research, MIT, MA, USA) [18], which offers a web interface that allows searching for potential miR-483-5p targets. Like other available methods, TargetScan relies on a variety of biochemical and biophysical features of the miRNA sequence, target mRNA sequence and its surrounding molecular context. Additionally, directly related to miR-483-5p, the TargetScan search indicates that the results include false positives, even though it provides no clear guidance on what specific details may contribute to this (beyond a generic statement regarding absolute and relative abundances of miRNA and mRNA within cells of interest). Some progress has been made by generating novel methods for evaluating miRNA:target binding, such as incorporation into a Variational Bayesian Gaussian mixed model approach [58] and adaptation for examining the differential binding to alternative alleles [59]. This has brought significant scope to extend the base TargetScan algorithm for specific purposes. What does remain clear though, is that experimental validation such as in our study is still necessary to properly establish the role of miRNAs in the repression of mRNA translation. Our experimental data were in line with the in silico predictions that ERK1 is repressed by miR-483-5p more efficiently than is ERK2.

The other important aspect to the identification of miRNA:mRNA interactions is the prior demonstration of interactions in published literature. To assist with this process, a number of tools have been developed that provide curated databases of experimentally-demonstrated miRNA:mRNA interactions. These tools have been reviewed in detail elsewhere [60], where four different systems were evaluated, including miRTarBase, and considerable variation was identified. The gold standard for the illustration of miRNA:target interactions is widely accepted to be the luciferase reporter assay, which demonstrates a direct interaction between target and miRNA. This indicates that our results provided strong evidence for the direct interaction between miR-483-5p and ERK1 (MAPK3) mRNA. There are a number of avenues in which this promising discovery could, and should, be expanded, namely; whilst we clearly demonstrate the downstream effect of miR-483-5p on TAU, it remains an open question whether there exists an additive impact directly on Aβ processing, however, it does have a context++ score of −0.33 with BACE2. This study also supports further research on the miR-483-5p/ERK1 interaction in primary neuronal culture and in vivo animal models. 

## 4. Materials and Methods

### 4.1. Cell Culture and Transfection

All cell lines were purchased from the American Type Culture Collection (ATCC, Manassas, VA, USA) except neonatal human dermal fibroblasts (HDFN) (No. DFM021711A, Zen Bio, Durham, NC, USA). Human embryonic kidney 293 (HEK293) cells, neuroblastoma SK-N-MC cells and neonatal human dermal fibroblasts were grown in Eagle’s Minimum Essential Medium (EMEM) (Sigma-Aldrich, St. Louis, MO, USA). EMEM was supplemented with 10 or 15% fetal bovine serum (FBS) (Sigma-Aldrich, St. Louis, MO, USA) (for HEK293 and SK-N-MC respectively), 2 mM l-glutamine and 100 mg/mL penicillin/streptomycin. For HDFN, EMEM was supplemented with 15% FBS, 2 mM l-glutamine and 1× antibiotic–antimycotic (all from Gibco, Grand Island, NY, USA). Cells were cultured at 37 °C in a humidified atmosphere of 5% CO_2_. Synthetic miRNA mimics were purchased: miR-483-5p mirVana miRNA mimic and mirVana miRNA mimic negative control (Ambion, Austin, TX, USA). mirVana miRNA mimic negative control (referred to as scramble) is a random sequence miRNA mimic molecule that has been extensively tested in human cell lines validated to not produce identifiable effects on known miRNA function. In miRNA transfection experiments, mirVana miRNA mimic negative control was used as baseline for evaluating the effect of miR-483-5p mimic. In these experiments, cells were seeded at 1 × 10^6^ cells/well in 12-well plates 24 h prior to transfection. Cells were grown to 60–80% confluence and transfected with miRNA mimics (up to 100 nM) alongside 100 ng of GFP plasmid (pEGFP-C1 from Addgene, Watertown, MA, USA). Cells were harvested at 48 h and 72 h post-transfection for the quantitative real-time polymerase chain reaction (RT-qPCR) and immunoblotting, respectively. A total of 100 ng of pEGFP-TAU plasmid (Addgene, Watertown, MA, USA) was transfected in HEK293 for TAU expression. Transfections in HEK293 and SK-N-MC were performed with Lipofectamine 2000 (Thermo Fisher Scientific, Waltham, MA, USA). In CRISPR/Cas9-mediated knockout experiments neonatal fibroblasts were seeded at 3 × 10^5^ cells/well in 6-well plates and grown to 80% confluence. Cells were transfected with constructs (250 ng of each plasmid, 500 ng of DNA in total) using Lipofectamine 3000 transfection reagent (Thermo Fisher Scientific, Waltham, MA, USA). Empty plasmids with Cas9 coding sequence, without sgRNAs, were used as a control. All transfections were performed with Opti-MEM medium and incubated for 6 h at 37 °C. Then, transfection media were replaced by fresh complete media and cells incubated for 72 h. For the ERK1/2 inhibition, SK-N-MC cells were treated with 10 µM U0126 (Sigma-Aldrich, St. Louis, MO, USA) or DMSO vehicle control (Sigma-Aldrich, St. Louis, MO, USA) for 48 h prior to immunoblot analysis. 

### 4.2. Single Guide RNA (sgRNA) Design and Cloning

sgRNAs targeting hsa-miR-483 were designed with the ‘Design and analyse guides’ tool on the Benchling platform (http://benchling.com, last accessed: 21 March 2021). To excise the sequence coding hsa-miR-483, Cas9 targets were designed both upstream and downstream of the gene. Sequences of upstream pairs of oligonucleotides were: sg1 forward—5′ CACCGAGCAGGAGCCCCATCACGG 3′; sg1 reverse—5′ AAACCCGTGATGGGGCTCCTGCTC 3′; and downstream pairs of oligonucleotides were: sg2 forward—5′ CACCGCTGCTGAGACAGGGAGACAA 3′; sg2 reverse—5′ AAACTTGTCTCCCTGTCTCAGCAG 3′. Forward and reverse oligonucleotides were annealed with T4 enzyme (Thermo Scientific, Waltham, MA, USA) according to the manufacturer’s instructions. Annealed sg1 and sg2 were cloned into pSpCas9(BB)-2A-GFP (PX458) (#48138, Addgene, Watertown, MA, USA) and pU6-(BbsI) CBh-Cas9-T2A-mCherry (#64324, Addgene, Watertown, MA, USA) plasmids, respectively, both digested with BbsI (Thermo Fisher Scientific, Waltham, MA, USA). *E. coli* (strain Mach1) was used to transform the plasmids, under ampicillin resistance (100 µg/mL). Constructs were confirmed by Sanger sequencing (Applied Biosystems, Bedford, MA, USA). 

### 4.3. Sorting and Genotyping of CRISPR/Cas9 Knocked-Out Neonatal Fibroblasts

Fibroblasts with efficient genomic deletion by both sgRNAs 1 and 2, i.e., co-expressing GFP and mCherry, were sorted on FACS Aria II (BD Biosciences, Franklin Lakes, NJ, USA). Forward scatter area (FSC-A) vs. side scatter area and FSC-A vs. FSC-width plots were used to exclude dead cells and doublets, respectively. An autofluorescence control was used to establish the positive gate. In total, 1–2 × 10^3^ cells were sorted into a single well of a 48-well plate and left recovering for 2 weeks. When cells reached about 90% confluence, they were transferred to 24-well plates and 10 µL of culture was used for genotyping confirmation of hsa-miR-483 knock-out. Cells were boiled, incubated with 1 µg/µL proteinase K at 56 °C and genomic deletion ascertained by PCR with the primers: forward, 5′-GCACGGCTTATTCCACCTGA-3′; reverse, 5′-ACAGAGAGAGCCGTGTTAGC-3′. The PCR products were analyzed in a 1.5% agarose gel. 

### 4.4. RNA Isolation and RT-qPCR

In miRNA mimic transfection experiments, total RNA was isolated from cells using the High Pure RNA isolation kit (Roche, Basel, Switzerland), according to the manufacturer’s instructions. RNA was quantified spectrophotometrically and its quality was analyzed using A260/A280 ratio in a BioPhotometer D30 (Eppendorf, Framingham, MA, USA). A total of 18S and 28S rRNA integrity was verified by electrophoresis in 1.5% agarose gels. RNA was then treated with DNase I and 20 ng of RNA was used to obtain cDNA using the High-Capacity cDNA Reverse Transcription Kit (Thermo Fisher Scientific, Waltham, MA, USA). Quantitative PCR was performed on an ABI 7900HT fast real-time PCR system (Applied Biosystems, Bedford, MA, USA). Briefly, cDNA (1:10 dilution of 20 ng), TaqMan Gene Expression Assays and TaqMan Universal Master Mix II (Thermo Fisher Scientific, Waltham, MA, USA) used with a thermal profile of an initial 10 min melting step at 95 °C, followed by 40 cycles at 95 °C for 15 s and 60 °C for 60 s. The relative fold change of ERK1 mRNA in the miR-483-5p transfected cells vs. scramble was normalized to GAPDH mRNA by 2^−ΔΔCt^ method [61]. In CRISPR knockout experiments, both miRNA and mRNA were isolated separately with Quick-RNA Microprep Kit (Zymo Research, Irvine, CA, USA) and were reverse transcribed with TaqMan^®^ Advanced miRNA Assays Single-tube assays (Applied Biosystems, Bedford, MA, USA), according to the manufacturers’ protocols, using a Veriti 96-Well Thermal Cycler (Applied Biosystems, Bedford, MA, USA). Briefly, 100 ng of mRNA was used to obtain cDNA and 1:10 dilution was used to obtain cDNA from miRNA. Quantitative PCR was performed according to manufacturer’s protocol using a TaqMan^®^ Fast Advanced Master Mix (Applied Biosystems, Bedford, MA, USA) with a thermal profile of 95 °C for 20 s; 95 °C for 3 s and 60 °C for 30 s in 40 cycles on a CFX96 Touch Real time PCR (Bio-Rad, Hercules, CA, USA). miR-191-5p was used as reference for miR-483-5p quantification and GAPDH was used as reference for mRNA quantification. The relative fold change of miR-483-5p and ERK1 in the CRISPR/Cas9-deleted fibroblast cells vs. controls was calculated using the 2^−ΔΔCt^ method [61]. Identification numbers of used probes: 477952_mir, hsa-miR-191-5p; 478432_mir, hsa-miR-483-5p; Hs00385075_m1, ERK1; Hs03929097_g1, GAPDH. 

### 4.5. Immunoblotting

Cellular lysates were obtained using RIPA lysis buffer (Sigma Aldrich, St. Louis, MO, USA) with phosphatase and protease inhibitor cocktails (Roche, Basel, Switzerland). The protein concentrations in the samples were determined using the Pierce BCA protein assay kit (Thermo Fisher Scientific, Waltham, MA, USA). A total of 20 µg of protein samples diluted in sample buffer containing 2-mercaptoethanol (Sigma-Aldrich, St. Louis, MO, USA), denatured for 10 min at 95 °C, were loaded in each well and separated by sodium dodecyl sulfate-polyacrylamide gel electrophoresis (SDS-PAGE) in a 10% polyacrylamide gel cast using the TGX FastCast acrylamide kit (Bio-Rad, Hercules, CA, USA). After electrophoretic separations, proteins were transferred to polyvinylidene fluoride (PVDF) membrane using a transfer unit (Bio-Rad, Hercules, CA, USA) at 80 V for 1 h. Uniformity of sample loading and the integrity of transfer was verified by staining with Ponceau-S (Sigma-Aldrich, St. Louis, MO, USA). After transfer, the PVDF membranes were blocked with 5% bovine serum albumin (BSA) for 2 h and incubated overnight at 4 °C with primary antibodies. These include: anti-ERK1/2 (Cell Signaling Technology, Danvers, MA, USA, #9102), anti-pERK1/2-pThr202/Tyr204 (Cell Signaling Technology, Danvers, MA, USA, #4370), anti-TAU (Dako, Santa Clara, CA, USA, #A0024) and anti-pTAU pSer199/Ser202 (Merck, Darmstadt, Germany, #AB9674) and anti-β-actin (Cell Signaling Technology, Danvers, MA, USA, #8H10D10). Detection of the immunoreaction was performed with a Clarity western enhanced chemiluminescence substrate kit (Bio-Rad, Hercules, CA, USA) using ChemiDoc MP imaging system (Bio-Rad, Hercules, CA, USA). 

### 4.6. Luciferase Reporter Assay

Plasmid constructs containing the predicted binding site for miR-483-5p were generated in pmirGLO dual-luciferase miRNA target expression vector (Promega, Madison, WI, USA). This vector is based on Promega’s dual-luciferase technology, with firefly luciferase (*luc2*) as the primary reporter for monitoring miRNA mediated regulation and renilla luciferase (*hRluc-neo*) as a secondary reporter for normalization. Specific oligonucleotides with DraI and XbaI ends containing single predicted binding site for miR-483-5p in the TAU 3′-UTR were synthesized in the laboratory of DNA sequencing and oligonucleotides synthesis in the Institute of Biochemistry and Biophysics (IBB), Warsaw, Poland (oligo.ibb.waw.pl, accessed: 1 March 2021). Oligonucleotide sequences of TAU 3’-UTR wild type (WT): forward—5′ AAATCCTTCCCACGGCCACTGCAGTCACCCCGTCTGCGCCGCTGTT 3′, reverse—5′ CTAGAACAGCGGCGCAGACGGGGTGACTGCAGTGGCCGTGGGAAGGATTT 3′; TAU 3’-UTR Mutation (MUT): forward—5′ AAATCCTTCCCACGGCCACTGCAGTCACAACATATGCGCCGCTGTT 3′, reverse—5′ CTAGAACAGCGGCGCATATGTTGTGACTGCAGTGGCCGTGGGAAGGATTT 3′; TAU 3’-UTR perfect match (PM): forward—5′ AAATCCTTCCCACCTCCCTTCTTTCCTCCCGTCTTGCGCCGCTGTT 3′, reverse—5′ CTAGAACAGCGGCGCAAGACGGGAGGAAAGAAGGGAGGTGGGAAGGATTT 3′. The appropriate oligonucleotides were annealed by boiling and gradual cooling, subsequently phosphorylated and cloned into the pmirGLO vector digested with DraI and XbaI restriction enzymes (New England Biolabs, Ipswich, MA, USA) at a multiple cloning site region downstream of the *luc2* gene. For miR-483-5p binding site in TAU (3′-UTR), three types of reporter plasmid constructs were prepared. Wild-type (WT) plasmid construct contains unaltered sites in the ligated binding site of TAU 3’-UTR as predicted. Negative control (CTR) consisted of transfection with the unmodified pmirGLO vector. Carrying mutations (MUT) plasmid construct contains mutated sites in the ligated binding site of TAU 3′-UTR. Perfect match (PM) plasmid construct contains full complementarity with the entire range of miR-483-5p in the ligated binding site of TAU 3′-UTR. All synthesized integrated sites (WT, MUT, PM) have 10-nucleotide flanking sequences, as recommended [62]. In all experiments, miR-483-5p and reporter constructs (WT/MUT/PM/CTR) were co-transfected. The luciferase activity was measured using a dual-luciferase reporter assay system (Promega, Madison, WI, USA) according to the manufacturer’s instructions with a Tecan infinite M-1000 Pro luminometer plate reader (Tecan, Maennedorf, Switzerland). In brief, HEK293 cells were harvested, washed with 1 × PBS and then were lysed in a passive lysis buffer (Promega, Madison, WI, USA). To these lysates, in 96-well plates, 100 µL of luciferase assay reagent was added. After briefly shaking, the light intensity was measured using the plate reader (Tecan, Maennedorf, Switzerland). 

### 4.7. Software and Statistical Analysis

To retrieve predicted targets and experimentally-validated targets for miR-483-5p, we used TargetScan v7.2 and miRTarBase v7.0, respectively [18,19]. Immunoblot densitometry was performed using Fiji software [63]. All statistical analyses were performed with R software v3.6.2 (https://cran.r-project.org/, accessed: 10 March 2021). For RT-qPCR, immunoblot and luciferase experiments, either unpaired student t test or one-way ANOVA with post–hoc Tukey HSD test as appropriate. *p* value < 0.05 was considered statistically significant, with plot annotations made only for data meeting that threshold, and the data are shown as mean ± standard error or mean ± standard deviation as indicated.

## 5. Conclusions

Further investigation of the miR-483-5p regulatory function in the ERK1/TAU axis will elucidate the molecular pathways underlying AD pathology and holds therapeutic potential against AD and other tauopathies. There are two primary obstacles between demonstration of the effect of miR-483-5p on TAU expression and its effective exploitation as an anti-tauopathic therapy: a deeper understanding of the interactome of miR-483-5p, including the potential for co-delivery with complementary miRNAs, and an optimal, specific delivery vehicle capable of crossing the blood-brain barrier to target specific cell/tissue types. 

## Data Availability

The data presented in this study are available in article and supplementary.

## Supplementary Materials

The following are available online at https://www.mdpi.com/article/10.3390/ijms22073653/s1, Figure S1: Context++ scores for common targets of miR-132-3p and miR-483-5p. Figure S2: Immunoblot original sources for figures.
