# Supplementary material for: Candidate Alzheimer’s Disease Biomarker miR-483-5p Lowers TAU Phosphorylation by Direct ERK1/2 Repression
Source: Int J Mol Sci. 2021 Apr 1;22(7):3653. doi: 10.3390/ijms22073653 (PMC8037306; doi:10.3390/ijms22073653)
Supplement: Supplementary file 1 [file ijms-22-03653-s001.zip › supplementary materials/Supplementary Figure S1.pdf]

A.

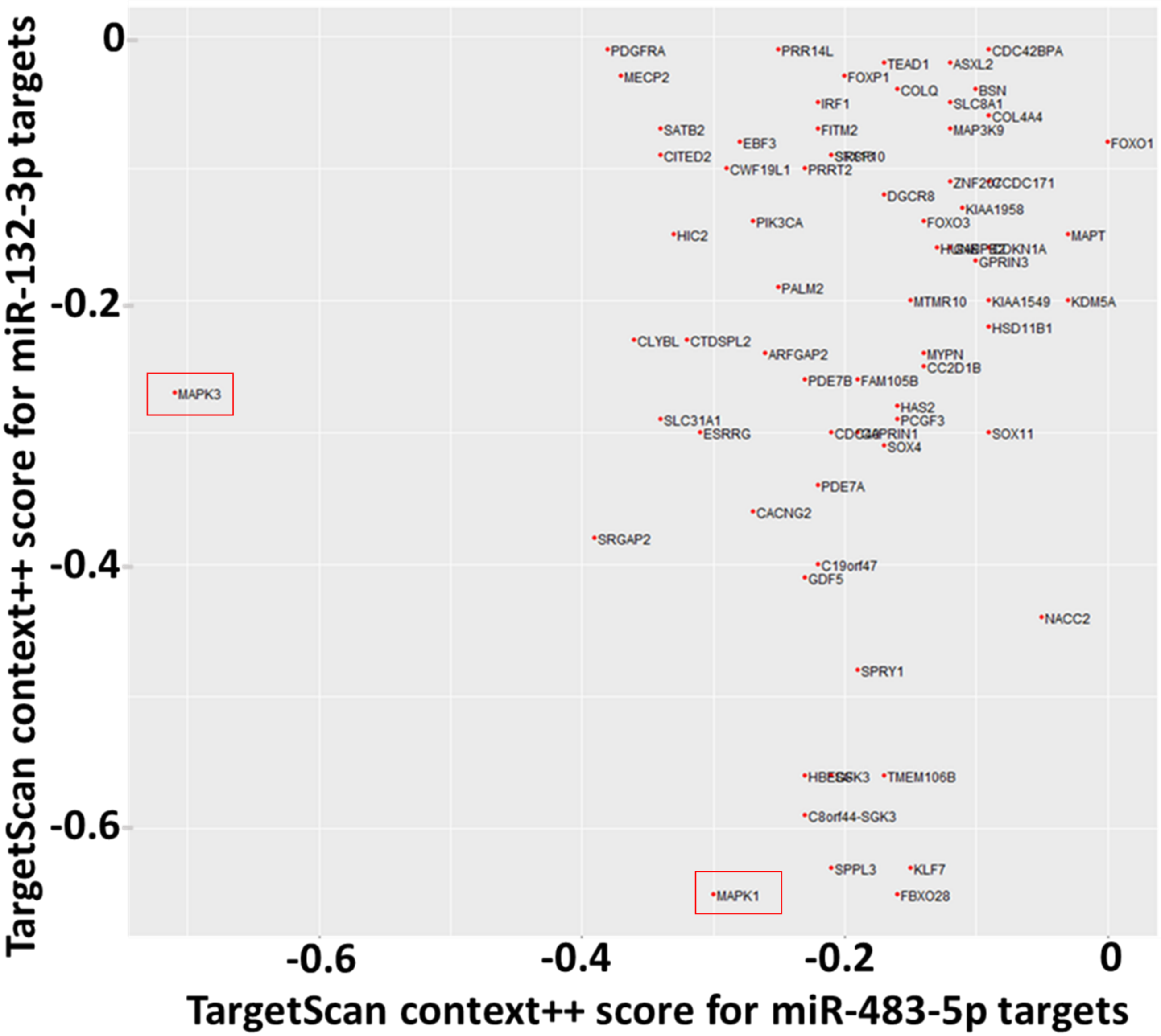

TargetScan context++ score for miR-132-3p targets
TargetScan context++ score for miR-483-5p targets
0
-0.2
-0.4
-0.6
-0.6
-0.4
-0.2
0
PDGFRA
PRR14L
CDC42BPA
TEAD1
ASXL2
MECP2
FOXP1
COLQ
BSN
IRF1
SLC8A1
COL4A4
SATB2
FITM2
MAP3K9
EBF3
FOXO1
CITED2
CWF19L1
PRRT2
DGCR8
KIAA1958
PIK3CA
FOXO3
HIC2
MAPT
GPRIN3
PALM2
MTMR10
KIAA1549
KDM5A
HSD11B1
CLYBL
CTDSPL2
ARFGAP2
MYPN
CC2D1B
MAPK3
PDE7B
FAM105B
HAS2
SLC31A1
PCGF3
ESRRG
SOX11
SOX4
PDE7A
CACNG2
SRGAP2
C19orf47
GDF5
NACC2
SPRY1
TMEM106B
C8orf44-SGK3
SPPL3
KLF7
MAPK1
FBXO28

B.

	Targets	Total context++ score for miR-483-5p targets	Total context++ score for miR-132-3p/212-3p targets
1	MAPK3	-0.71	-0.27
2	PDGFRA	-0.38	-0.01
3	SRGAP2	-0.39	-0.38
4	SATB2	-0.34	-0.07
5	HIC2	-0.33	-0.15
6	CTDSPL2	-0.32	-0.23
7	ESRRG	-0.31	-0.3
8	CITED2	-0.34	-0.09
9	ARFGAP2	-0.26	-0.24
10	PRR14L	-0.25	-0.01
11	MAPK1	-0.3	-0.65
12	SLC31A1	-0.34	-0.29
13	GDF5	-0.23	-0.41
14	PRRT2	-0.23	-0.1
15	HBEGF	-0.23	-0.56
16	CWF19L1	-0.29	-0.1
17	EBF3	-0.28	-0.08
18	CDC40	-0.21	-0.3
19	C8orf44-SGK3	-0.23	-0.59
20	PALM2	-0.25	-0.19
21	SRSF10	-0.21	-0.09
22	SGK3	-0.21	-0.56
23	SOX4	-0.17	-0.31
24	TEAD1	-0.17	-0.02
25	STX16	-0.21	-0.09
26	DGCR8	-0.17	-0.12
27	COLQ	-0.16	-0.04
28	FITM2	-0.22	-0.07
29	KLF7	-0.15	-0.63
30	FOXO3	-0.14	-0.14
31	PCGF3	-0.16	-0.29
32	FAM105B	-0.19	-0.26
33	CC2D1B	-0.14	-0.25
34	HUNK	-0.13	-0.16

35	MAP3K9	-0.12	-0.07
36	BSN	-0.1	-0.04
37	CDKN1A	-0.09	-0.16
38	GPRIN3	-0.1	-0.17
39	GABPB2	-0.12	-0.16
40	CDC42BPA	-0.09	-0.01
41	COL4A4	-0.09	-0.06
42	FBXO28	-0.16	-0.65
43	KIAA1549	-0.09	-0.2
44	SPPL3	-0.21	-0.63
45	MECP2	-0.37	-0.03
46	ZNF207	-0.12	-0.11
47	NACC2	-0.05	-0.44
48	MYPN	-0.14	-0.24
49	ASXL2	-0.12	-0.02
50	KIAA1958	-0.11	-0.13
51	MAPT	-0.03	-0.15
52	PIK3CA	-0.27	-0.14
53	SOX11	-0.09	-0.3
54	MTMR10	-0.15	-0.2
55	CLYBL	-0.36	-0.23
56	PDE7B	-0.23	-0.26
57	C19orf47	-0.22	-0.4
58	IRF1	-0.22	-0.05
59	SLC8A1	-0.12	-0.05
60	KDM5A	-0.03	-0.2
61	CCDC171	-0.09	-0.11
62	FOXP1	-0.2	-0.03
63	HAS2	-0.16	-0.28
64	TMEM106B	-0.17	-0.56
65	CACNG2	-0.27	-0.36
66	HSD11B1	-0.09	-0.22
67	PDE7A	-0.22	-0.34
68	SPRY1	-0.19	-0.48
69	CAPRIN1	-0.19	-0.3
70	FOXO1	0	-0.08

Supplementary figure 1. (A) 70 common predicted targets for miR-483-5p and miR-132-3p/212-3p are plotted based on the total context++ score from TargetScan(v7.2) (B) Total context++ score values for 70 common predicted targets for miR-483-5p and miR-132-3p/212-3p were shown
