# Supplementary material for: Candidate Alzheimer’s Disease Biomarker miR-483-5p Lowers TAU Phosphorylation by Direct ERK1/2 Repression
Source: Int J Mol Sci. 2021 Apr 1;22(7):3653. doi: 10.3390/ijms22073653 (PMC8037306; doi:10.3390/ijms22073653)
Supplement: Supplementary file 1 [file ijms-22-03653-s001.zip › supplementary materials/Supplementary_Source_figures.pdf]

Supplementary figures

Article

Candidate Alzheimer's Disease Biomarker miR-483-5p Lowers TAU Phosphorylation by Direct ERK1/2 Repression

Siranjeevi Nagaraj [1], Andrew Want [1], Katarzyna Laskowska-Kaszub [1], Aleksandra Fesiuk [1,2], Sara Vaz [2], Elsa Logarinho [2,3] and Urszula Wojda [1,*]

[1] Laboratory of Preclinical Testing of Higher Standard, Nencki Institute of Experimental Biology of Polish Academy of Sciences, Pasteur 3, 02-093 Warsaw, Poland; s.nagaraj@nencki.edu.pl (S.N.); a.want@nencki.edu.pl (A.W.); k.laskowska-kaszub@nencki.edu.pl (K.L.-K.); a.fesiuk@nencki.edu.pl (A.F.)

[2] i3S, Institute for Research and Innovation in Health, University of Porto, 4200-135 Porto, Portugal; sara.vaz@ibmc.up.pt (S.V.); elsa.logarinho@ibmc.up.pt (E.L.)

[3] Aging and Aneuploidy Laboratory, IBMC, Institute of Molecular and Cellular Biology, University of Porto, 4200-135 Porto, Portugal

* Correspondence: u.wojda@nencki.edu.pl; Tel.: +48-22-5892578

Original images for figure 3,5,6,7 are provided. In each figure we provide detailed annotation of full figure matching the figure descriptions in the manuscript followed by raw unlablled orginal images [order of the unlablled orginal images shown: raw image without protein marker , greyscale image with protein marker and colur image with protein marker]

<u>Detailed Information about immunoblot analysis of figure 3D.</u>

Lane 1 2 3 shows transfection with scramble and lane 4 5 6 shows transfection with miR-483-5p

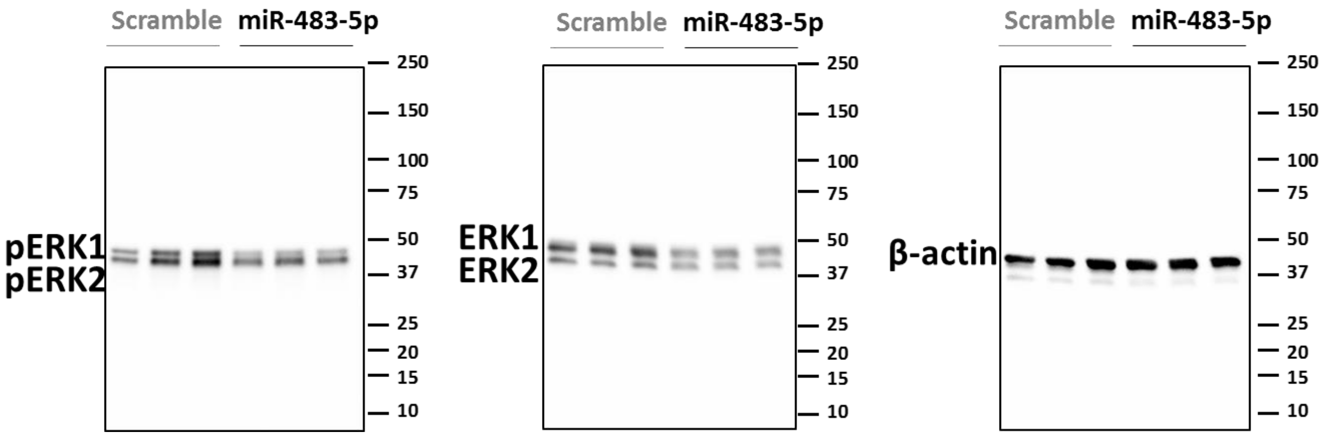

Unlabelled raw blots[Uncropped, untouched , full original images of immunoblots] are shown below

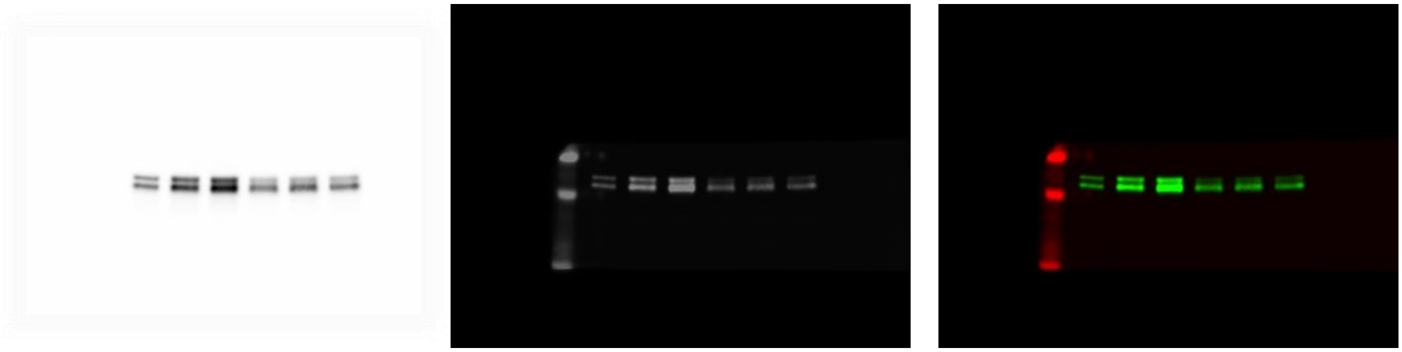

pERK 1/2

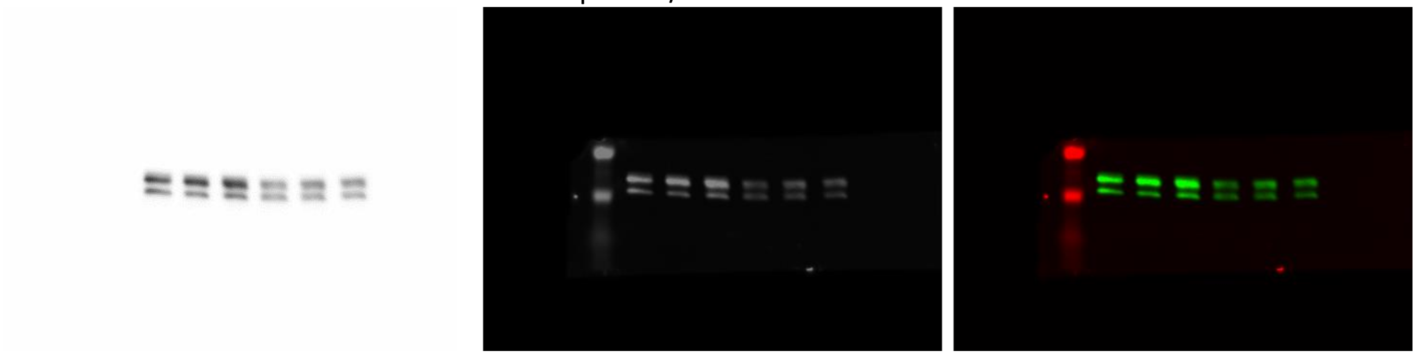

ERK1/2

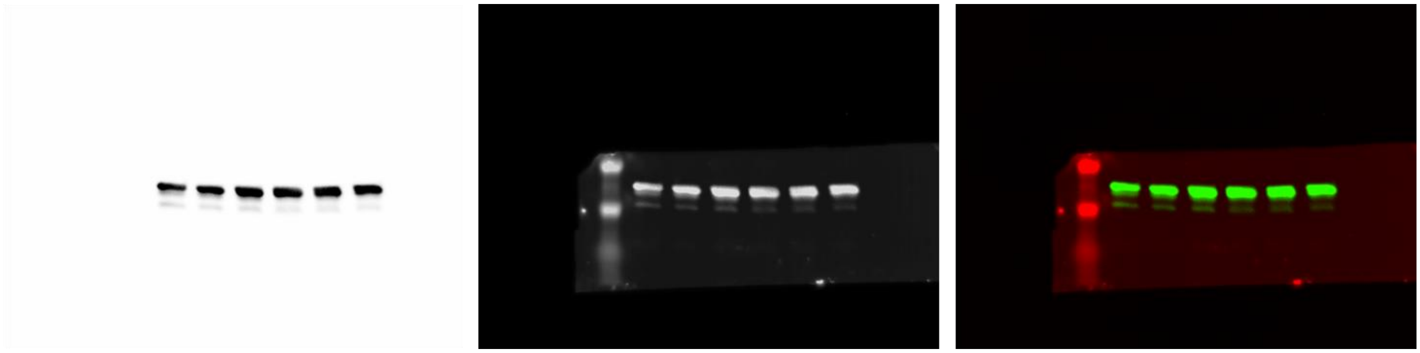

β-actin

Detailed Information about immunoblot analysis of figure 5.

Lane 1 2 3 shows transfection with scramble and lane 4 5 6 shows transfection with miR-483-5p

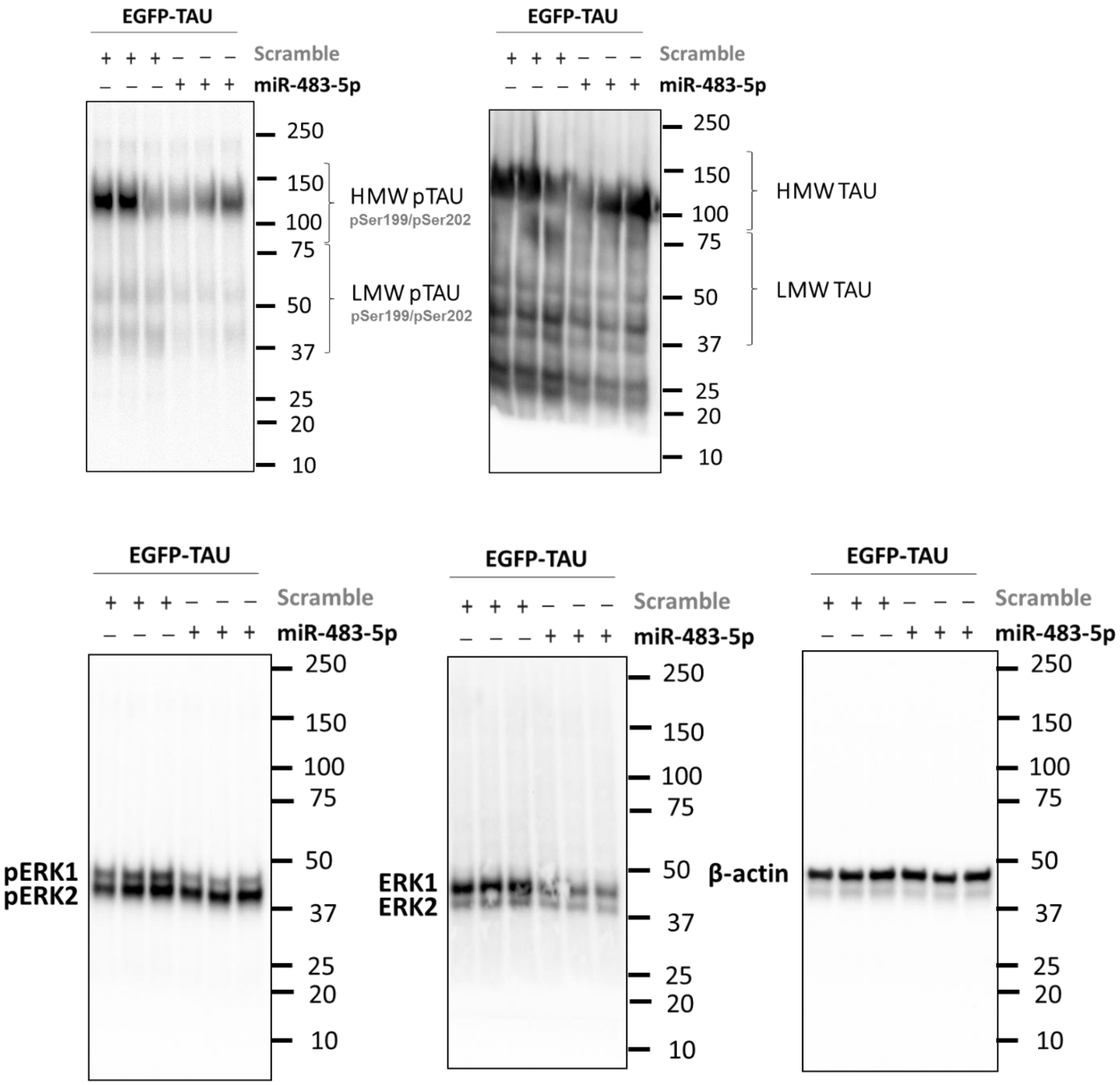

Unlabelled raw blots[Uncropped, untouched , full original images of immunoblots] are shown below (First 3 out of 9 lanes are omitted for analysis in figure 5 from the raw blot because it is HEK293 cells without EGFP-TAU)

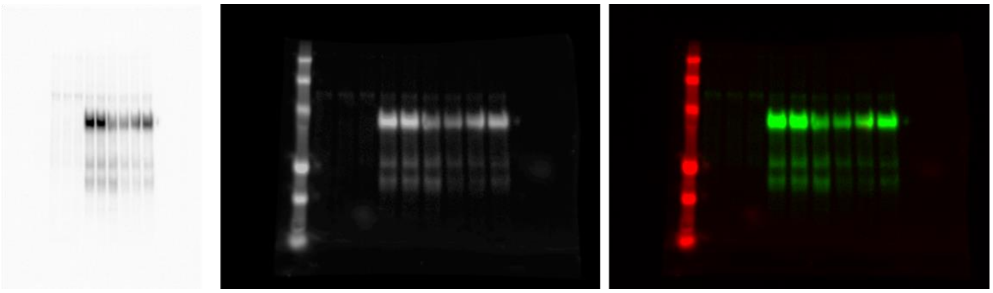

pTAU

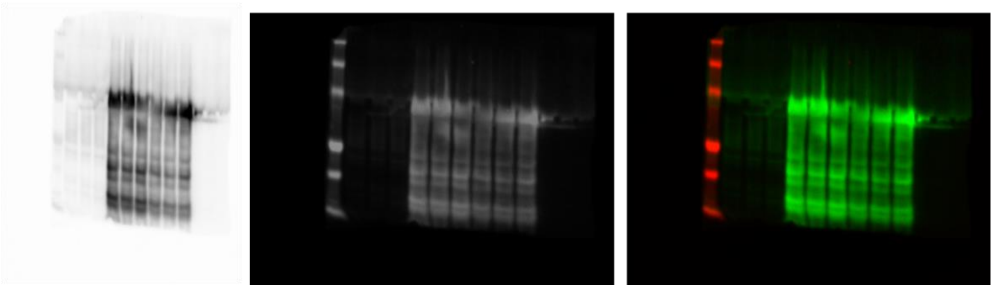

TAU

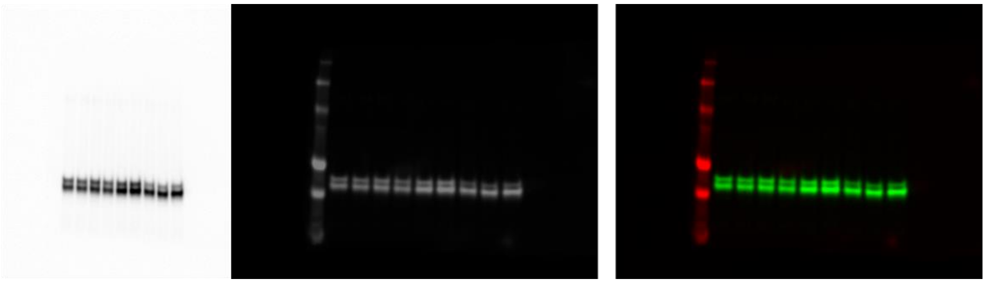

pERK1/2

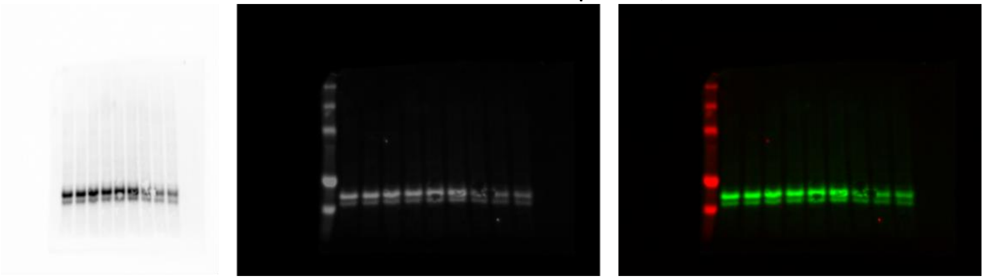

ERK1/2

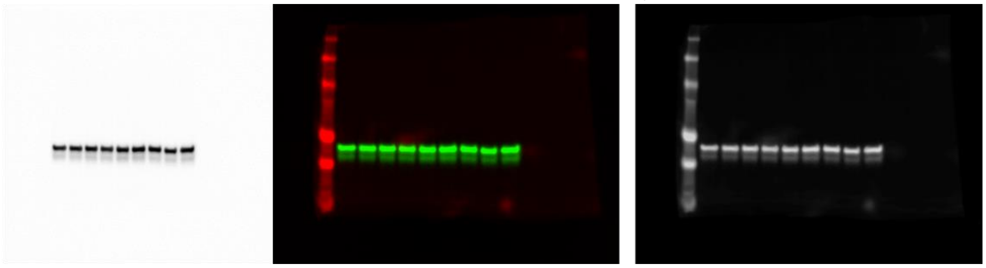

β-actin

Detailed Information about immunoblot analysis of figure 6.

Lane 1 2 3 shows treatment with DMSO and lane 4 5 6 shows treatment with U0126

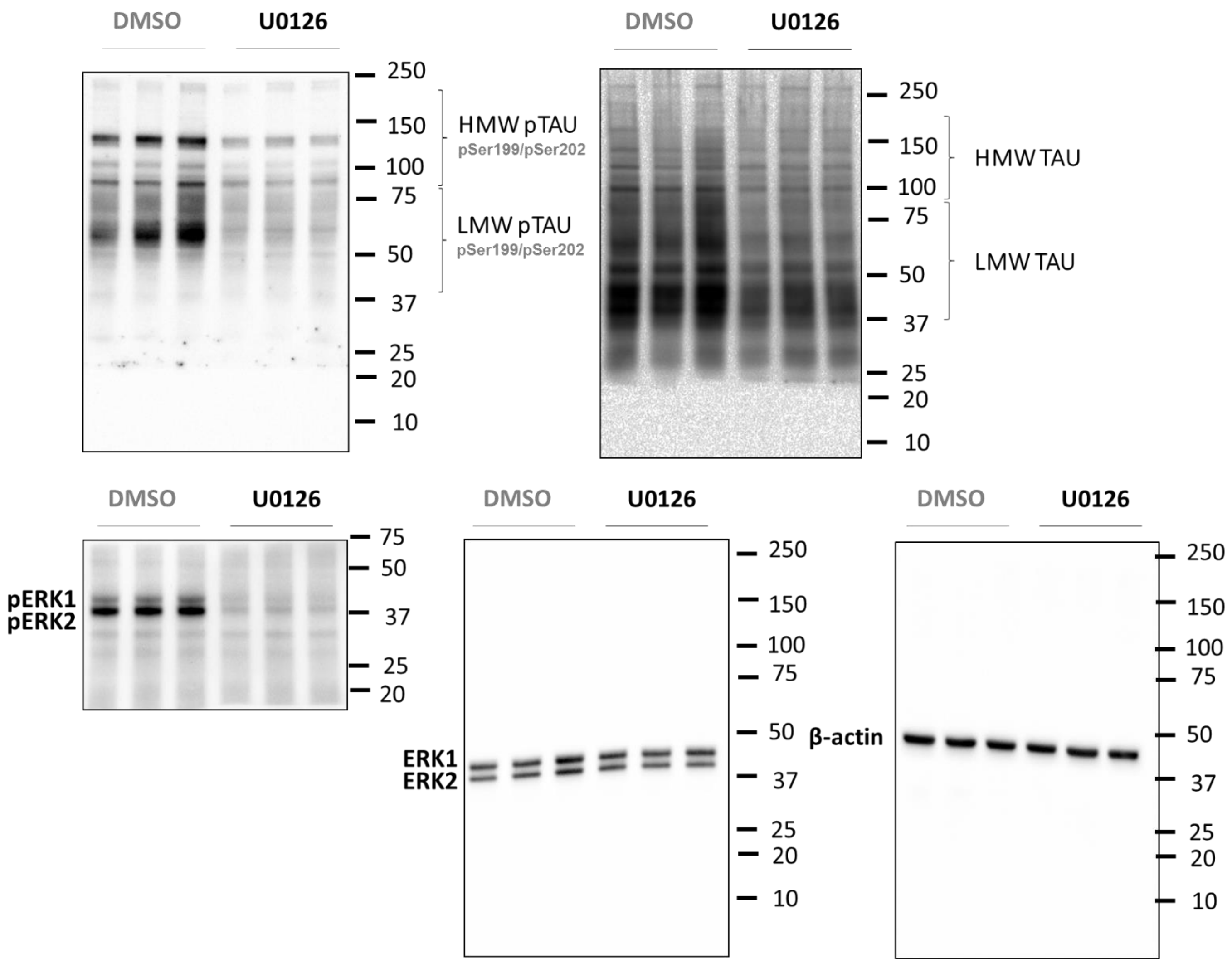

Unlabelled raw blots[Uncropped, untouched , full original images of immunoblots] are shown below

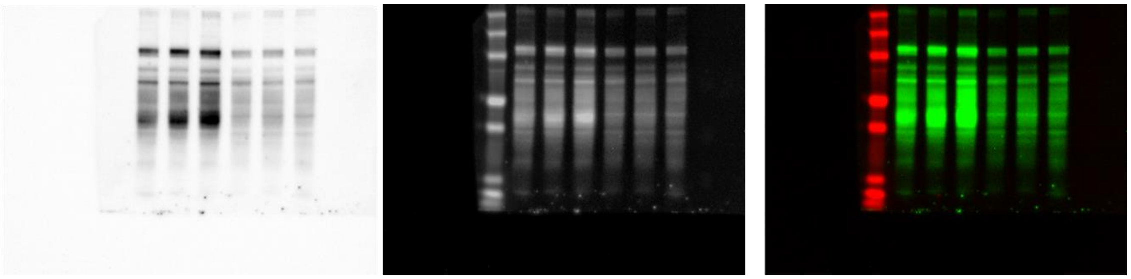

pTAU

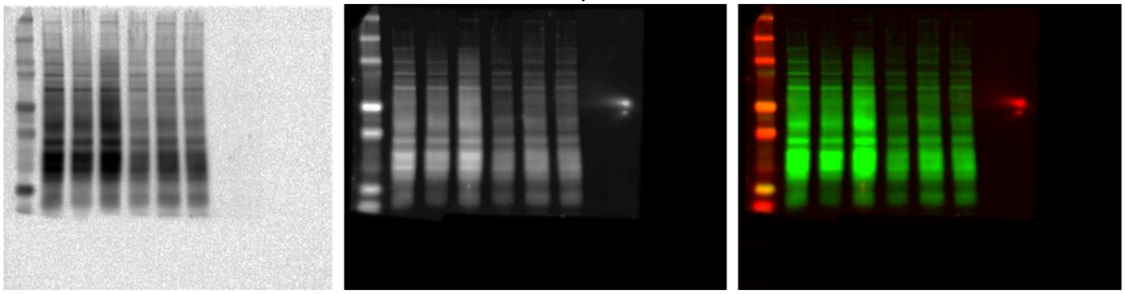

TAU

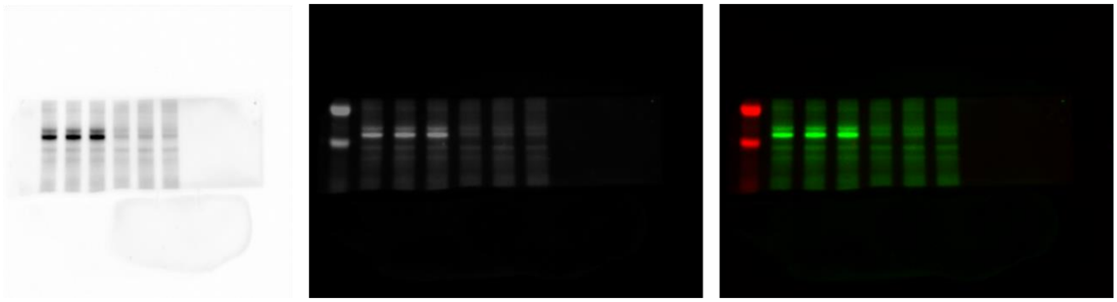

pERK1/2

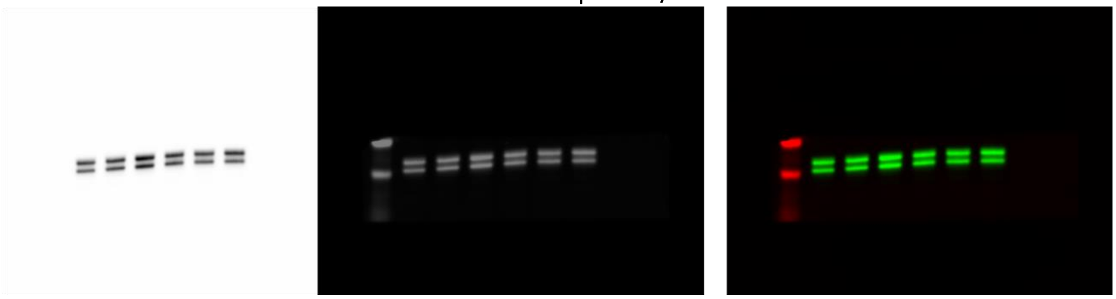

ERK1/2

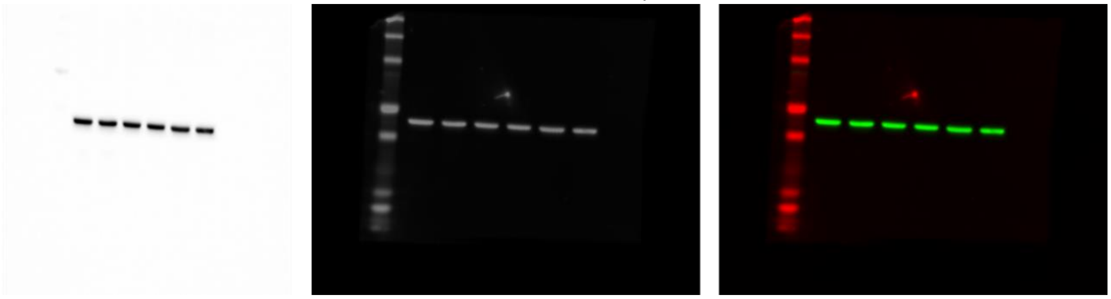

β-actin

Detailed information about immunoblot analysis of figure 7.

Lane 1 2 shows mock transfection and lane 3 4 shows transfection with miR-483-5p

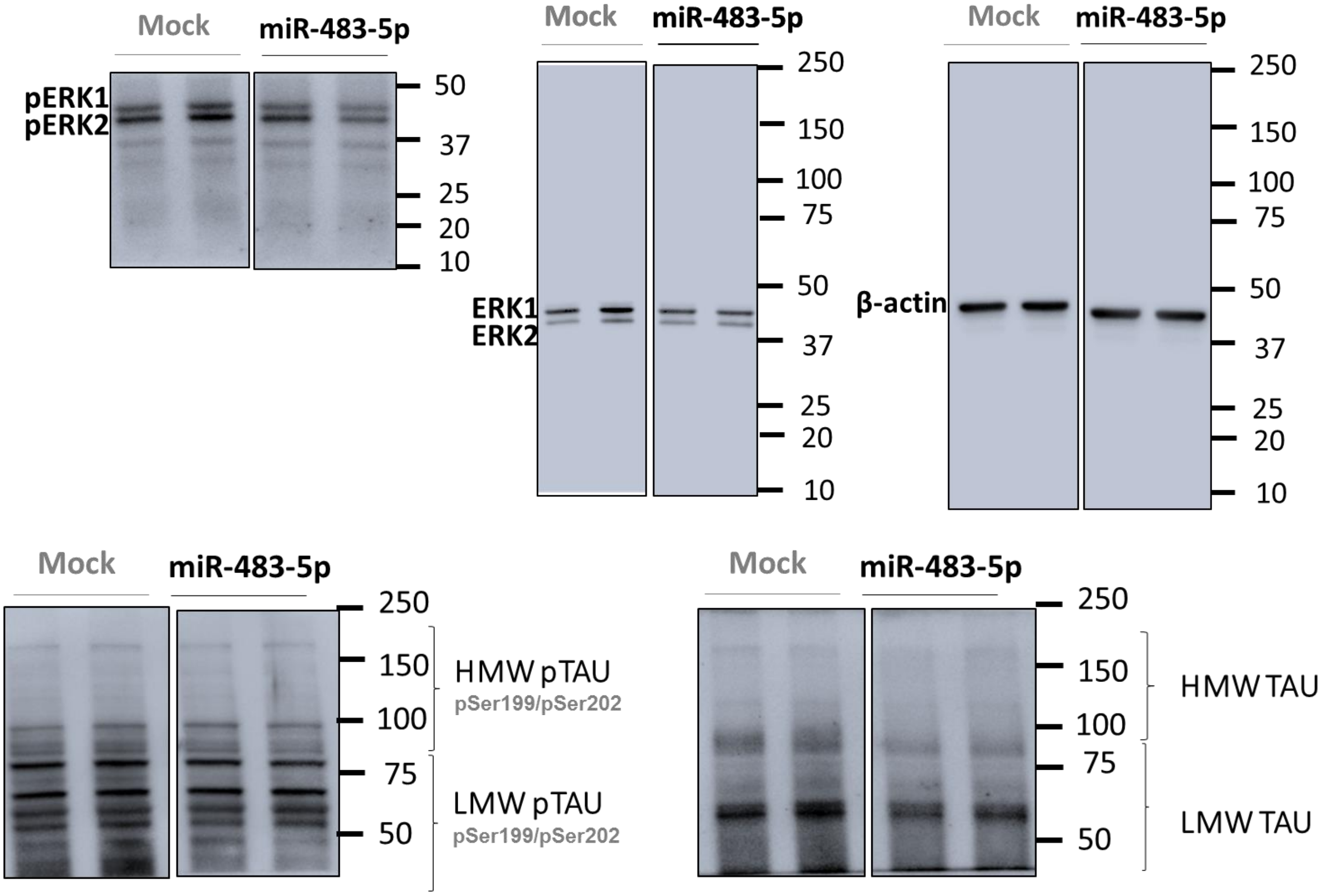

Unlabelled raw blots[Uncropped, untouched , full original images of immunoblots] are shown below
(To show representative cases 2,3 lanes for Mock and 5,6 lanes for miR-483-5p were used in Figure 7)

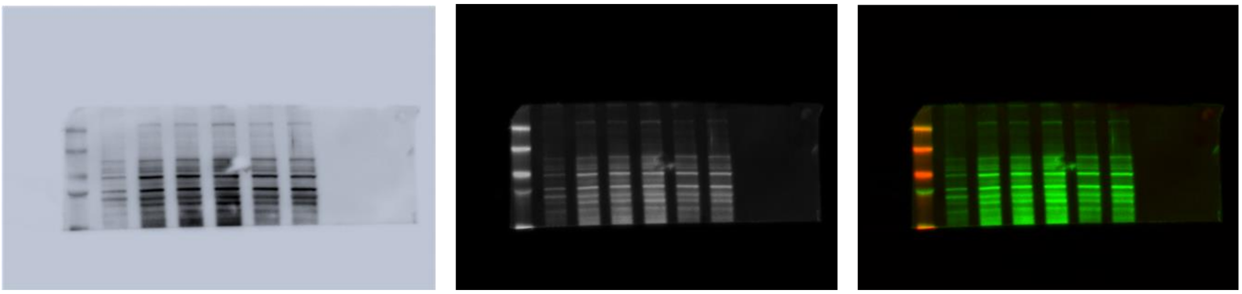

pTAU

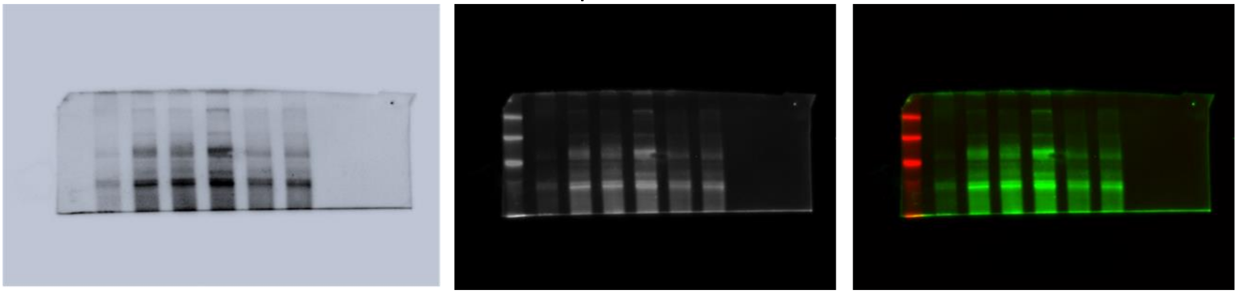

TAU

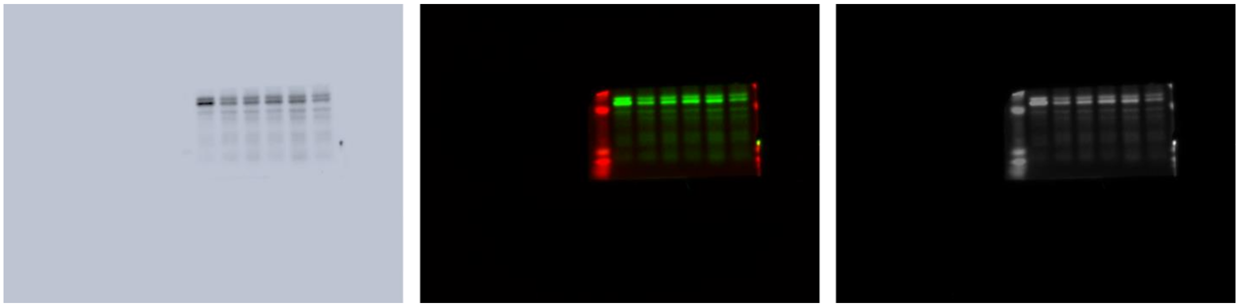

pERK1/2

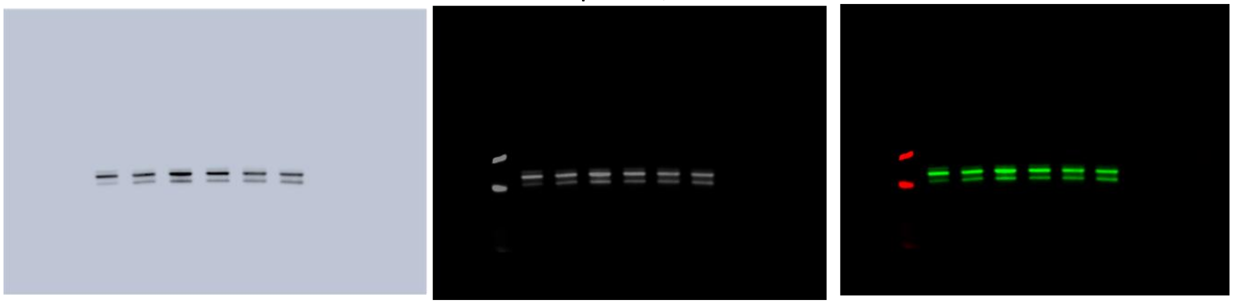

ERK1/2

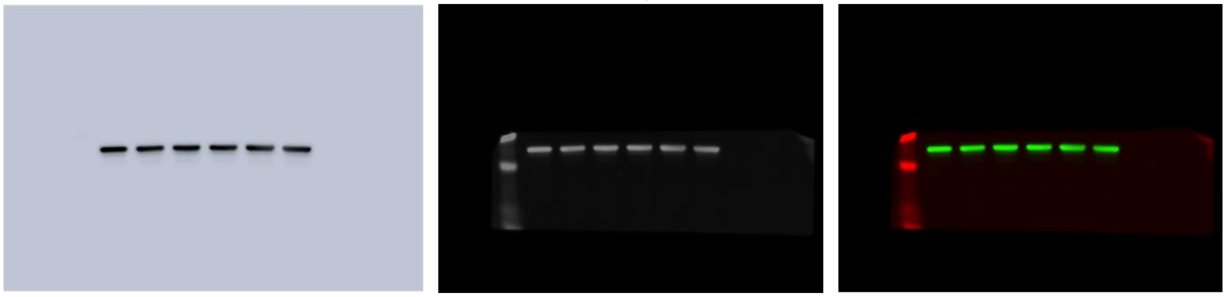

β-actin
